# A Zero-Touch Dynamic Configuration Management Framework for Time-Sensitive Networking (TSN)

**DOI:** 10.3390/e27060584

**Published:** 2025-05-30

**Authors:** Junhui Jiang, Shanyu Jin, Xinghan Li, Kaisong Zhang, Baodan Sun

**Affiliations:** College of Computer Science and Technology, Harbin Engineering University, Harbin 150001, China; jhjiang@hrbeu.edu.cn (J.J.);

**Keywords:** Industry 5.0, Time-Sensitive Networking, dynamic configuration management

## Abstract

As Industry 5.0 progresses, the demand for zero-touch configuration in industrial automation and smart manufacturing is increasing. This paper proposes a dynamic configuration management framework for Time-Sensitive Networking (TSN), aiming to address the challenges of flexibility and adaptability in dynamic network environments. A zero-touch configuration model is presented for TSN by incorporating a Delay-Aware Shortest Path Search (DASPS) algorithm to improve scheduling success rates. Simulation results demonstrate the ability of the framework to reconfigure networks within 2.67 milliseconds. The DASPS algorithm achieves a scheduling success rate of 70.22% for 1000 TSN flows, in contrast to only 22.23% achieved by the Shortest Path Search (SPS) algorithm. The proposed model effectively adapts to dynamic network changes, guaranteeing real-time data transmission. To further evaluate system adaptability, path entropy is introduced as a metric to quantitatively assess the balance of scheduling outcomes under topological changes. In the event of link failures, path entropy experiences a sharp decline but rapidly recovers after reconfiguration, demonstrating the system’s strong self-healing capability.

## 1. Introduction

Industry 5.0 combines human intelligence with advanced technologies to achieve human-centric, sustainable, and resilient manufacturing systems [1]. It requires the data to be transmitted in a real-time and deterministic manner. Time-Sensitive Networking (TSN) provides the high reliability and the low-latency traffic scheduling capabilities required in industrial scenarios and, based on a standard Ethernet architecture, offers a clear and practical solution for industrial interconnection [2].

TSN addresses such requirements via a set of IEEE 802.1 standards [3] to guarantee timely and predictable data delivery. TSN offers key features such as time synchronization [4], deterministic transmission [5], ultra-high reliability [6], and flexible configuration [7]. TSN is increasingly recognized as a pivotal communication technology for future industrial networks [8]. IEEE 802.1Qbv is a key component for TSN to achieve deterministic communication. It defines a Time-Aware Shaper (TAS) with eight queues and timed gates. TAS has received widespread attention for its ability to provide deterministic timing guarantees by leveraging network-wide synchronization and time-triggered scheduling mechanisms [9]. TAS is controlled by a Gate Control List (GCL), which opens specific gates at predetermined times to allow the transmission of designated traffic, ensuring deterministic transmission. Although the IEEE 802.1Qbv standard specifies the scheduling mechanism of TAS, clear best practices for GCL population and queue assignment at each hop are still lacking [10].

Qbv-oriented traffic scheduling methods can be primarily classified into satisfiability modulo theories (SMT) [11,12], integer linear programming (ILP) [13,14], and heuristic algorithms [15,16]. Chaine et al. [11] proposed the Egress-TT approach, consisting of Exclusive Queue Allocation and Size-Based Isolation. This approach simplifies upgrades and reduces computational effort. An SDN-based flow scheduling algorithm for conflict-free TSN was proposed in [12]; this algorithm reduces delays and ensures deterministic network performance. Craciunas and Oliver [13] addressed schedule robustness against synchronization loss in 802.1Qbv by ensuring deterministic frame behavior using a Z3 SMT/OMT-based scheduler tool. Feng et al. [14] proposed a proactive fault tolerance scheduling algorithm to enhance reliability by preemptively identifying and mitigating potential faults. Huang et al. [15] proposed an Earliest Window heuristic scheduling algorithm for in-vehicle networks; this algorithm uses an SMT solver for GCL calculation and ensures QoS for various traffic types. Zhang et al. [16] introduced a flow-aware NW-TAS scheduling algorithm, transforming the no-wait forwarding model to reduce latency and increase scheduling speed for TSN.

These methods typically formulate the scheduling problem with various constraints and then solve it using appropriate solvers. They assume that traffic paths are fixed or selected using the shortest path search (SPS) algorithm, ignoring the coupling relationship between traffic scheduling and path selection. The SPS focuses only on finding the shortest path between the source and destination, which could lead to network congestion on specific routes. This congestion increases the difficulty of traffic scheduling and reduces its success rate. Furthermore, dynamic network scenarios are crucial in Industry 5.0 due to the need for real-time adaptability and resilience. For example, in plug-and-produce systems for smart manufacturing, devices are dynamically added or removed, leading to changes in the network topology. Consequently, a dynamic configuration network management architecture is mandatory to handle these dynamic changes seamlessly.

However, existing TSN traffic scheduling methods are designed for static networks, where the GCL is computed before the system runs. Once the network undergoes dynamic changes, the precomputed GCL cannot guarantee real-time traffic transmission. Moreover, existing static network management frameworks cannot sense dynamic changes, such as topology and traffic variations, in real time. These are critical input parameters for path selection and traffic scheduling. In other words, network configuration cannot keep pace with dynamic changes, failing to satisfy the requirements for deterministic data transmission. To address these issues, we first propose a zero-touch configuration model for TSN. Next, we introduce a DASPS algorithm to avoid congestion caused by SPS. Finally, several simulation experiments are conducted to validate the effectiveness of our framework and algorithm. The key contributions are summarized as follows:

(1) A zero-touch configuration model is proposed to automatically detect network state changes, recalculate traffic paths and schedules, and configure these updates to network nodes in real-time.
(2) The proposed DASPS algorithm formulates the delay constraint as a maximum hop count constraint and assigns the flow to the path with the most remaining bandwidth.
(3) Simulation experiments demonstrate that the proposed framework can reconfigure the network in 2.67 milliseconds. The DASPS algorithm significantly improves scheduling success rates compared to the SPS algorithm. Specifically, DASPS achieves a 70.22% scheduling success rate for 1000 TSN flows, while SPS only achieves 22.23%.

## 2. Dynamic Configuration Management Framework

To address the challenge of dynamic network changes, we propose a dynamic configuration management framework. This framework includes a zero-touch configuration model for dynamically managing TSN systems and a DASPS algorithm to enhance the scheduling success rate.

### 2.1. Zero-Touch Configuration Model

**Figure 1:**
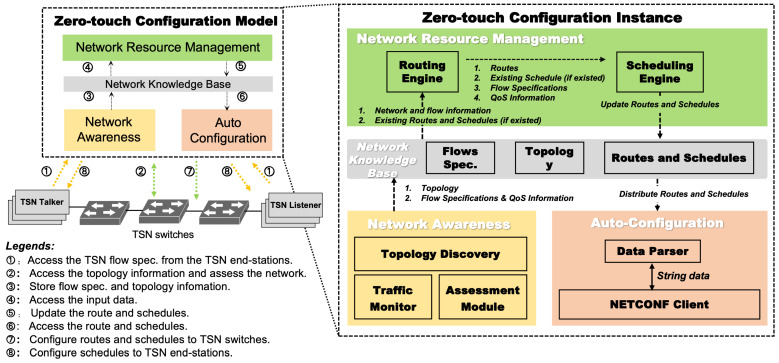
Zero-touch configuration model for TSN.

By referring to IEEE standard 802.1Qcc [7], we propose a zero-touch configuration model for TSN, as illustrated in Figure 1. The left side displays the overall structure, while the right side shows the detailed implementation of each module. In general, *Network Awareness* periodically checks the network state to quickly detect dynamic changes, such as topology or traffic parameter changes. The *Network Knowledge Base* stores the network state and triggers *Network Resource Management* to recalculate paths and GCL. Once the calculations are complete, *Auto Configuration* is activated to automatically configure the TSN end devices and switches. The implementation details are discussed in the following subsections.

*(1) Network Awareness:* We extend the central user configuration (CUC) to be *Network Awareness* to collect the network status, which contains *Topology Discovery*, a *Traffic Monitor*, and *Assessment Module*. The *Topology Discovery* module is designed and implemented based on the Link Layer Discovery Protocol (LLDP). *Topology Discovery* is implemented by periodically sending LLDP packets to neighboring devices and listening for incoming LLDP packets. These packets identify network connections. The data collected are processed to create a real-time network topology map, which is continuously updated based on the LLDP information received. The *Traffic Monitor* module uses packet sniffing techniques to monitor traffic flows and gather the necessary parameters, e.g., source, destination, period, and length. The *Assessment Module* evaluates network performance through end-to-end (E2E) delay. It captures timestamps at the source and destination of flow. The difference between these timestamps provides the E2E delay measurement to assess the real-time performance.

*(2) Network Knowledge Base:* It is implemented as an XML-based data table that stores traffic specifications, topology parameters, routing paths, and GCLs. This base is continuously updated to reflect the current state of the network, ensuring accurate and efficient recalculations of paths and schedules.

*(3) Network Resource Management:* It contains two modules: a *Routing Engine* for path selection and a *Scheduling Engine* for traffic scheduling. DASPS is employed as the *Routing Engine*, which will be detailed in Section 2.2. A traffic scheduling method called *TSM* is adopted for the *Scheduling Engine*, with details available in [17]. Historical paths and GCLs are fully utilized to recalculate new paths and schedules in response to network changes, thereby significantly reducing the calculation time.

*(4) Auto-configuration:* To achieve automatic configuration, it adopts NETCONF/YANG, consisting of two functions: a *Data Parser* and a *NETCONF Client*. The *Data Parser* accesses path and scheduling information, converting it into string format. The *NETCONF Client* is implemented using a NETCONF Python library. It configures paths and GCLs for TSN switches and end-stations through NETCONF/YANG. NETCONF is a standardized protocol for network device management, while YANG is a data modeling language for defining configuration data. NETCONF/YANG enables consistent and interoperable configuration across multi-vendor network devices.

### 2.2. DASPS Algorithm

The DASPS algorithm allocates paths with the maximum available bandwidth to flows while adhering to delay constraints to guarantee real-time transmission. As presented in Algorithm 1, the inputs to DASPS are TSN flow sets *F*, a directed graph $G^{i}$, and the processing delay ($d_{proc_{j}}$) of a TSN switch $SW_{j}$. The flow set *F* comprises multiple TSN flows $f_{i}$, each specified by length $l_{i}$, period $p_{i}$, source $src_{i}$, destination $dst_{i}$, and delay constraint $\varphi_{i}$. The network is modeled as a graph $G^{i}=\left(V,E\right)$, where *V* represents the set of TSN end-stations and switches, while *E* represents the set of all directional links between source $v_{i}$ and destination $v_{j}$, where $E=\{\left(v_{i},v_{j}\right)|v_{i},v_{j}\in V\}$. The output of the algorithm consists of the routing paths $P_{i}$.

The algorithm begins with lines 1 to 5, where all TSN flows are sorted in ascending order of their delay constraint $\varphi_{i}$ using the *SortByConstraint* function. Smaller $\varphi_{i}$ values denote tighter constraints. Sorting these flows first ensures that they are processed first, which improves the overall scheduling success rate. In line 6, the algorithm iterates over the sorted set of TSN flows, i.e., $F_{\mathrm{sorted}}$.

For each flow, the parameters are extracted using the *minPop* function. In line 8, the maximum allowable hop count ($MaxHop_{i}$) is calculated using the *CalMaxHop* function. It calculates $MaxHop_{i}$ for a given TSN flow by solving the following inequality:

(1) $$\varphi_{i}\geq D_{e2e}^{i}=\sum_{j=1}^{M+1}d_{\mathrm{trans}}+\sum_{j=1}^{M+1}d_{{\mathrm{prop}}_{j}}+\sum_{j=1}^{M}d_{{\mathrm{proc}}_{j}}+\sum_{j=1}^{M}d_{{\mathrm{queue}}_{j}}^{{\mathrm{SW}}_{j}}$$

where *M* denotes the number of switches between $src_{i}$ and $dst_{i}$. The end-to-end delay $D_{e2e}^{i}$ must be less than $\varphi_{i}$. Since the TSM algorithm eliminates the queuing delay, $\sum_{j=1}^{M}d_{{\mathrm{queue}}_{j}}^{{\mathrm{SW}}_{j}}$ is equal to zero. The processing delay $\sum_{j=1}^{M}d_{{\mathrm{proc}}_{j}}$ is given as input, and the propagation delay $\sum_{j=1}^{M+1}d_{{\mathrm{prop}}_{j}}$ and transmission delay $\sum_{j=1}^{M+1}d_{\mathrm{trans}}$ can be calculated using the link speed $S_{v_{i},v_{j}}$, link length $L_{v_{i},v_{j}}$, and flow length $l_{i}$.

With all variables known except *M*, setting $\varphi_{i}=D_{e2e}^{i}$ allows solving for the maximum value of *M*. This value represents the maximum allowable number of switches along the routing path, denoted as $MaxHop_{i}$. If the hop count exceeds this value, the end-to-end delay will surpass the constraint, in which case the real-time requirements cannot be guaranteed.

Lines 9 to 11 initialize essential data structures for the pathfinding stage.The number of nodes *n* in $G^{i}$ is determined, and the predecessor array is set up to keep track of the previous node for each node in the path. The priority queue, denoted as priorityQueue, is initialized with the source node, setting the initial distance and hop count to zero.
**Algorithm 1:** DASPS algorithm.
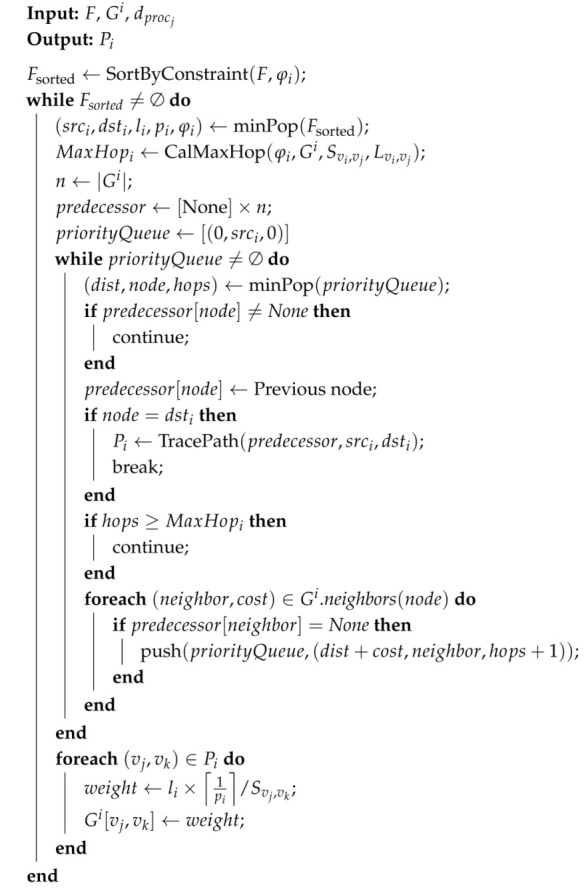


The main routing loop, defined in lines 12 to 23, performs a bounded best-first search. At each step, the node with the minimum cumulative path cost is extracted from the queue using *minPop*. If the node has already been visited, as indicated in the previous array, the algorithm proceeds to the next iteration. The predecessor of the current node is recorded to facilitate later path reconstruction.

When the current node is the destination $dst_{i}$, the path is reconstructed using the *TracePath* function, and the loop ends. If the hop count exceeds the maximum allowable hop count $MaxHop_{i}$, the algorithm continues to the next iteration. For each neighbor of the current node, if the neighbor has not been visited, it is added to the priority queue with the updated distance and hop count.

Once a valid path $P_{i}$ is determined, the algorithm updates the weights of all edges along this path, as described in lines 27 to 30. The weight update is crucial for optimizing bandwidth utilization for subsequent flows. The weight of each edge is calculated based on the flow length $l_{i}$, the period $p_{i}$, and the link speed $S_{v_{j},v_{k}}$. Specifically, the weight is computed and updated using the formula in line 28. This formula ensures that the link capacity is appropriately allocated according to the flow characteristics. The network graph $G^{i}$ is then updated with these new weights for the edges in the path, ensuring efficient bandwidth utilization.

By concurrently considering path selection and delay constraints, the DASPS algorithm reduces scheduling complexity and improves the overall scheduling success rate. The detailed evaluation will be presented in Section 3.

### 2.3. Configuration Procedure

**Figure 2:**
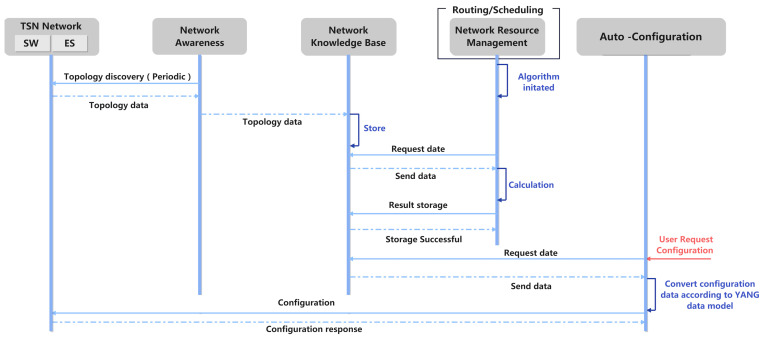
Sequencing of the configuration procedure.

The configuration work is completed by the Network Awareness, Network Knowledge Base, Network Resource Management, and Auto-configuration modules. Figure 2 shows the timing of the process. The solid blue arrows indicate information exchange between modules, while the dashed blue arrows represent internal self-generated messages. The solid red arrows denote user configuration requests. Initially, the Network Awareness module periodically monitors topology changes to perceive the current network status. Subsequently, the Network Knowledge Base stores the acquired topology data, serving as the foundation for path computation and scheduling recalculations. The Network Resource Management module employs the DASPS algorithm to select paths with the largest remaining bandwidth, while ensuring compliance with delay constraints and hop limits. It then performs traffic scheduling in conjunction with TSN mechanisms. Finally, the Auto-configuration module utilizes the NETCONF/YANG protocol to distribute the updated paths and GCLs to TSN switches and end-stations, achieving zero-touch configuration of network devices.

## 3. Implementation and Evaluation

### 3.1. System Implementation

The proposed zero-touch configuration model was implemented using the OMNeT++ simulator. We adopted the INET framework to implement the four key components of the configuration framework, i.e., *Network Awareness*, *Auto-configuration*, *Network Knowledge Base*, and *Network Resource Management*.

*Network Awareness* contains three elements, wherein the *Topology Discovery* and *Traffic Monitor* elements were implemented by using the *cTopology* and *cPacket* objects, respectively. Such objects are provided by the official library. Timestamps were added to each packet at the source and destination nodes to enable accurate measurement of end-to-end delays. Thus, the end-to-end delay can be calculated by the *Assessment Module*.

We implemented the proposed DASPS algorithm to be the *Routing Engine*, and we directly adopted the TSM [17] to be the *Scheduling Engine*. The calculated routes and schedules are configured for the TSN simulation nodes by *Auto-configuration*. The key network parameters, such as flow specifications, topology, routes, and schedules, are contained in the *Network Knowledge Base*.

### 3.2. Numerical Results

To validate the proposed configuration framework and DASPS, the Barabási–Albert model generated a random topology consisting of TSN switches and end-stations. The simulation environment is summarized in Table 1. The link speed and length were configured to 1 Gbps and 100 m. All the flow lengths were set to the MTU size. The source and destination of flows were randomly selected among the TSN end-stations. The transmission intervals were randomly selected among 2 ms, 4 ms, 8 ms, and 16 ms. The delay constraint was equal to the transmission interval of a flow. The experiments used Python 3.12 to generate random topologies, with subsequent simulations in OMNeT++ 6.0.2. The simulations were performed on a PC featuring an Intel i9-12900 CPU and 32GB of RAM.

Firstly, 10 sets of simulations were conducted with a number of TSN switches ranging from 10 to 55. Each set had a random topology, with the number of flows ranging from 10 to 100 in increments of 10, resulting in 100 tests. Table 2 presents the results for the scenario with 10 switches and 10 flows, showing that none of the actual hop counts exceeded the maximum hop count. The results of other experiments consistently demonstrated that the DASPS algorithm met the maximum hop count constraint when selecting flow paths. Due to space constraints, detailed results from other experiments are not included.

**Figure 3:**
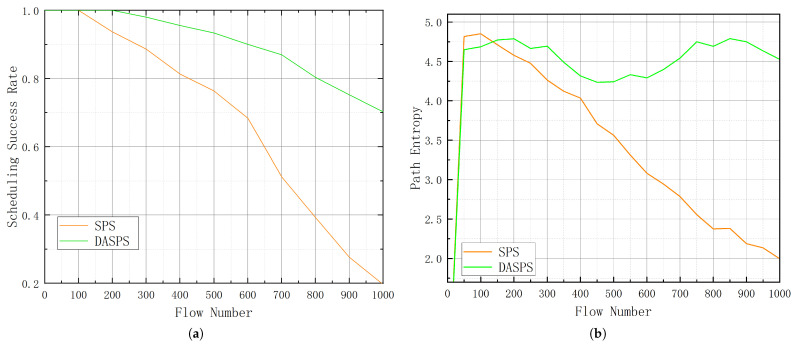
Comparison of scheduling success rate and path entropy between DASPS and SPS. (**a**) Scheduling success rate; (**b**) Path entropy variation.

The TSN traffic scheduling success rate is the ratio of successfully scheduled traffic flows to the total number of flows within a specified time constraint. Such a metric is critical to guarantee the real-time and deterministic capabilities. The path searching algorithm is coupled with the traffic scheduling algorithm, since it significantly affects the scheduling success rate. Hence, the scheduling success rate was adopted to assess the proposed DASPS, which is compared to that of a pure shortest path searching algorithm (SPS). As mentioned, we adopted TSM to schedule traffic while searching the paths using DASPS and SPS, respectively. NetworkX was employed to generate the random topology with 30 TSN switches. In Figure 3a, the Y-axis presents the scheduling success rate, while the X-axis denotes the number of flows, ranging from 50 to 1000, with a step size of 50. The orange and green lines show the results of SPS and DASPS, respectively. Figure 3b shows that when the number of flows increases, the path entropy of DASPS is higher than that of SPS, reaching a maximum of 6.21, while SPS is always lower than 5.3, which means that SPS is more likely to cause path concentration.

When the number of flows is within 100, both DASPS and SPS maintain a 100% scheduling success rate. However, when the number of flows exceeds 100, the scheduling success rate of SPS continuously declines. In particular, when the number of flows exceeds 600, the scheduling success rate drops sharply to 22.23%. DASPS significantly improves the scheduling success rate of the scheduling algorithm. Even if 1000 flows are input, the scheduling success rate can still be maintained at 70.22%. The only goal of SPS is to find the shortest path; the link load is not considered, which causes congestion of one link and significantly reduces the scheduling success rate. DASPS transforms the delay constraint into a hop constraint and selects the path with the largest remaining bandwidth while satisfying the hop constraint. Thus, network congestion is avoided, and the scheduling success rate is improved. The numerical results show that DASPS can significantly enhance the success rate of scheduling under high-load conditions, thus ensuring real-time data transmission.

**Figure 4:**
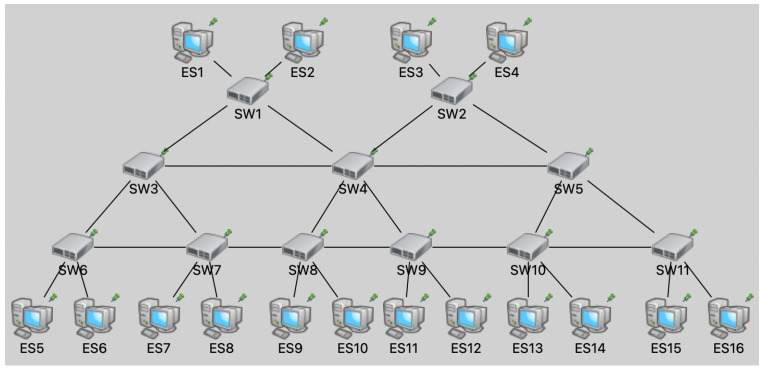
Simulation topology.

**Figure 5:**
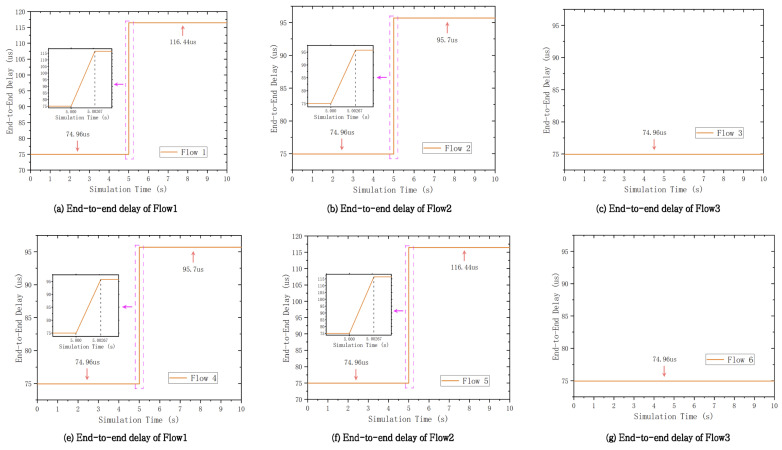
End-to-end delay results.

To validate the reconfiguration performance, the dynamic simulation scenario is established by randomly disconnecting or connecting network nodes. We adopt a typical industrial control mesh topology as shown in Figure 4, which contains 11 TSN switches interconnected in pairs. The links are configured with a length of 100 m and a rate of 1 Gbps. TSN end-stations are distributed to the switches, which can send or receive the TSN traffic. This scenario contains six TSN flows with the same configuration specifications as mentioned above. The scenario is implemented using OMNeT++ and is managed by the proposed configuration framework. At the fifth second of the simulation run, the network undergoes a dynamic change through the disconnection of SW3 and SW5. The proposed configuration framework detects the topology change and rapidly triggers the routing and scheduling engines to reallocate resources. The recalculations are then automatically configured to the network nodes. Table 3 shows the routing paths of six flows before and after network failure, corresponding to the “Pre” and “Post” rows, respectively, under the “Fault Status” column. The paths of Flow1, Flow2, Flow4, and Flow5 have changed. The disconnection of SW3 and SW5 resulted in these nodes being excluded from the paths of these flows. However, the paths of Flow3 and Flow6 remained unaffected by the failure of these nodes. In addition, we measured the end-to-end delay (E2E) for these six flows as shown in Figure 5. The X-axis represents the simulation run time in seconds, and the Y-axis represents the E2E delay in microseconds. Each flow was assigned a randomly selected delay constraint ranging from 2 to 16 milliseconds. The E2E delays in the figure range from hundreds of microseconds to well below their respective delay constraints, thus satisfying real-time performance requirements. The paths of Flow3 and Flow6 were not affected by node failures, resulting in nearly constant E2E delays. In contrast, Flow1, Flow2, Flow4, and Flow5 experienced abrupt changes in the E2E delay at the 5-s mark due to node failures. These delays stabilized at higher values after 2.67 milliseconds, primarily due to increased path hop counts. The durations of topology discovery and network configuration were set to 1 millisecond each, with the computation of routing and scheduling tables taking only 670 microseconds. The results demonstrate the capability of the proposed framework for rapid reconfiguration. It effectively adapts to dynamic network changes and enables the reconstruction and reconfiguration of flow paths and scheduling lists.

**Figure 6:**
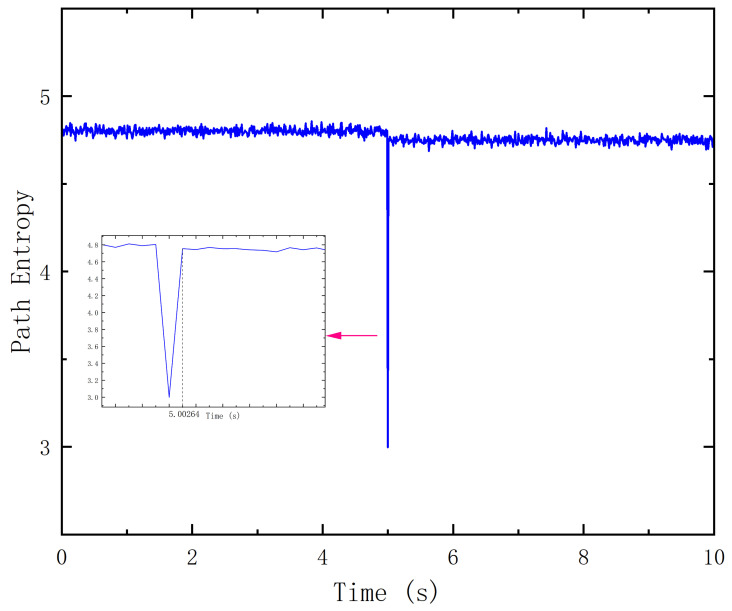
Path entropy under link failure.

Figure 6 shows the trend of dynamic change in path information entropy during link disconnection and the automatic reconfiguration process in the network. The experimental scenario is based on the typical industrial control network topology, as shown in Figure 4. At the fifth second of the simulation, the connections between SW3 and SW6 and between SW5 and SW10 are deliberately severed to induce topology changes. During the experiment, link load data are collected every 0.1 s, and the path entropy is calculated, with a time span of 0 to 10 s. The experimental results show that when a fault occurs, the path entropy value drops from about 4.8 to 3.0, reflecting the phenomenon of scheduling path concentration and network load imbalance. After the reconfiguration mechanism was started, the entropy value rebounded and returned to near the initial level after 2.64 milliseconds, showing an efficient recovery trend. The experiment verifies that the proposed framework has excellent fast response and path reconfiguration capabilities in dynamic network environments.

## 4. Conclusions

This paper presents a method for managing dynamic network changes in Industry 5.0 using a zero-touch configuration model for Time-Sensitive Networking (TSN). The proposed model can detect network changes and reallocate resources accordingly, guaranteeing real-time data transmission. The proposed DASPS algorithm is designed to find paths with the most remaining bandwidth while satisfying delay constraints. This approach improves scheduling success rates, achieving 70.22% for 1000 TSN flows compared to 22.23% with the SPS algorithm. The simulation results show the ability of the framework to reconfigure networks in 2.67 milliseconds, maintaining consistent scheduling success rates under varying loads. During link failures, path entropy drops significantly and rapidly recovers after reconfiguration, demonstrating strong self-healing capability. The proposed model and algorithm enhance the flexibility, scalability, and reliability of TSN-based industrial networks. Future research will focus on developing deep reinforcement learning (DRL)-based algorithms to further optimize the framework. 

## Figures and Tables

**Table 1:** Simulation environment configuration.

Parameter	Value
Link Speed	1 Gbps
Link Length	100 m
Python Version	3.12
CPU	Intel Core i9-12900
RAM	32 GB

**Table 2:** Hop count analysis.

Flow Name	Max. Hop Count	Actual Hop Count
Flow1	4	3
Flow2	3	2
Flow3	6	3
Flow4	7	5
Flow5	6	4
Flow6	3	2
Flow7	9	6
Flow8	8	5
Flow9	7	5
Flow10	8	3

**Table 3:** Network flow path analysis.

Flow Name	Fault Status	Path
Flow1	Pre	ES1, SW1, SW3, SW6, ES5
	Post	ES1, SW1, SW4, SW8, SW7, SW6, ES5
Flow2	Pre	ES2, SW1, SW3, SW7, ES7
	Post	ES2, SW1, SW4, SW8, SW7, ES7
Flow3	Pre	ES1, SW1, SW4, SW9, ES11
	Post	ES1, SW1, SW4, SW9, ES11
Flow4	Pre	ES3, SW2, SW5, SW10, ES13
	Post	ES3, SW2, SW4, SW9, SW10, ES13
Flow5	Pre	ES4, SW2, SW5, SW11, ES15
	Post	ES4, SW2, SW4, SW9, SW10, SW11, ES15
Flow6	Pre	ES4, SW2, SW4, SW8, ES9
	Post	ES4, SW2, SW4, SW8, ES9

## Data Availability

The raw data supporting the conclusions of this article will be made available by the authors on request.
